# Effects of Increasing Concentrations of Sodium Sulfite on Deoxynivalenol and Deoxynivalenol Sulfonate Concentrations of Maize Kernels and Maize Meal Preserved at Various Moisture Content

**DOI:** 10.3390/toxins7030791

**Published:** 2015-03-09

**Authors:** Marleen Paulick, Inga Rempe, Susanne Kersten, Dian Schatzmayr, Heidi Elisabeth Schwartz-Zimmermann, Sven Dänicke

**Affiliations:** 1 Institute of Animal Nutrition, Friedrich-Loeffler-Institute (FLI), Federal Research Institute for Animal Health, Bundesallee 50, 38116 Braunschweig, Germany; E-Mails: marleen.paulick@fli.bund.de (M.P.); inga.rempe@gmx.de (I.R.); susanne.kersten@fli.bund.de (S.K.); 2 Biomin Holding GmbH, Biomin Research Center, Technopark 1, 3430 Tulln, Austria; E-Mail: dian.schatzmayr@biomin.net; 3 Christian Doppler Laboratory for Mycotoxin Metabolism and Center for Analytical Chemistry, Department for Agrobiotechnology (IFA-Tulln), University of Natural Resources and Life Sciences, Vienna, Konrad Lorenz Str. 20, 3430 Tulln, Austria; E-Mail: heidi.schwartz@biomin.net

**Keywords:** decontamination, deoxynivalenol, deoxynivalenol sulfonates, sodium sulfite, wet preservation, maize

## Abstract

Under moderate climatic conditions, deoxynivalenol (DON) contamination occurs frequently on cereals. Detoxification measures are required to avoid adverse effects on farm animals. In the present study, a wet preservation method with sodium sulfite (Na_2_SO_3_) and propionic acid was tested to titrate the optimum Na_2_SO_3_-dose for maximum DON reduction of contaminated maize kernels and meal and to examine the interaction between dose and moisture content in dependence on the preservation duration. The DON concentration decreased with increasing amounts of supplemented Na_2_SO_3_ and with increasing duration of the preservation period in a bi-exponential fashion. Additionally, the feed structure and moisture content had a significant influence on the decontaminating effect. Variants with 30% moisture content favored higher DON reduction rates compared to 14% moisture, but especially at low moisture contents, DON reduction was more pronounced in maize kernels than in maize meal. In addition to the decrease of DON, a concomitant formation of three different DON sulfonates was observed which differed in their formation pattern over the time course of preservation. The overall results and statistical analysis clarified that Na_2_SO_3_ addition of 10 g/kg maize at 30% moisture for eight days was necessary to obtain a complete DON reduction.

## 1. Introduction

The occurrence of moulds on agricultural crops, entailing serious consequences for plant cultivation and also farm animal production, is an extensively-studied research area [1,2]. The temperate climate of Central Europe particularly favors the proliferation of the genus Fusarium; its occurrence is usually accompanied by the formation of a variety of mycotoxins [3]. Deoxynivalenol (DON) and zearalenone are of special importance with regard to animal health as discussed by Döll and Dänicke (2011) [2]. Because of the toxin formation in the field, these mycotoxins are still present during storage of contaminated harvest products. Depending on dose, DON exposure might result in reduced feed intake, feed refusal or vomiting, especially in pigs, whereby the general growth performance is decreased [4,5]. For this reason, there is an ongoing need for possibilities to decontaminate mycotoxin-contaminated feedstuffs [2]. Several studies have shown that chemical treatment with sulfur-containing reagents such as sodium bisulfite [6,7,8] and sodium metabisulfite (SBS) [9,10] is suitable for the reduction of DON. During the reaction a sulfonate group is added to C10 of the DON molecule.

For our study, sodium sulfite as an alternative to SBS was chosen, indicating a DON-reducing effect similar to the other sulphur containing substances [11,12]. Depending on the pH value, three different DON sulfonates (DONS 1, 2 and 3) are formed but different relationships to each other could be found [11]. The efficiency for the degradation of DON was strongly influenced by the moisture content and the applied dose of the additive. Higher moisture content was used to reduce the amount of chemical used for a significant DON reduction.

To verify these hypotheses, DON contaminated maize was treated with increasing dosages of sodium sulfite at two different moisture levels and stored for 79 days in order to investigate the decontamination of DON containing maize by wet preservation with Na_2_SO_3_ and propionic acid and to evaluate that strategy for post-harvest reduction of DON contaminated cereals. Additionally, two different structures of maize (kernels, MK *vs.* meal, MM) were included to check their influence on the reaction because the comparison was not examined yet.

This strategy should serve the purpose to minimize the harmful effects of DON for livestock as far as possible and to comply with existing guidance values in farm animal feeding [13].

## 2. Results

In the experiment, maize kernels and meal were treated with sodium sulfite and propionic acid at two different moisture content levels and stored over 79 days. During the preservation time, samples were taken to determine the concentrations of DON and DON metabolites as well as to check the adjusted moisture content, the pH value and microbial status.

### 2.1. Moisture Content and pH-Value

The target moisture contents were 14% and 30%. The determination of dry matter confirmed the achievement of intended moisture contents (Table 1). The moisture content varied only marginally as indicated by the low standard deviations.

The pH values increased with increasing additions of Na_2_SO_3_ (Table 1). While the mean pH value without Na_2_SO_3_ addition was 4.58, the addition of 10 g Na_2_SO_3_/kg increased the pH value to 4.89. The dose related effect was not dependent on feed matrix or preservation duration.

### 2.2. Deoxynivalenol Concentration

**Figure 1:**
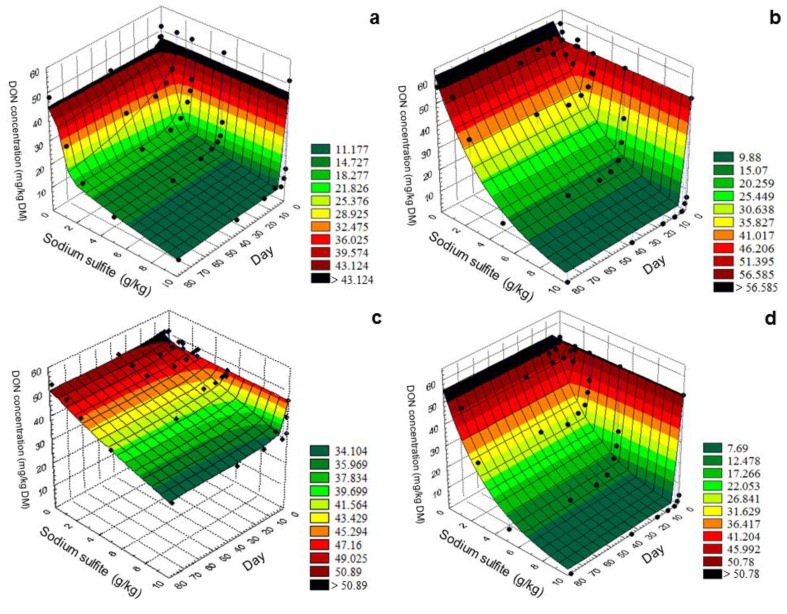
Bi-exponential regression of increasing dosages of supplemented sodium sulfite (Na_2_SO_3_; 0–10 g/kg) and of preservation time (0–79 days) on deoxynivalenol (DON) concentration of maize for various combinations of feed structure (MK = maize kernels, MM = maize meal) and moisture content (14% or 30%): (**a**) MK 14%; (**b**) MK 30%; (**c**) MM 14%; (**d**) MM 30% (see Equation (1) and Table 2 for regression details).

The kinetics of DON reduction indicated a steep initial decrease and was followed by a slow but steady decline, particularly at Na_2_SO_3_ addition ≥2.5 g/kg. Below this Na_2_SO_3_ dose, the DON concentration values pointed to a strong scattering, and no satisfactory DON reduction was achieved (Figure 1). In broad terms, the DON concentration decreased with increasing amounts of added Na_2_SO_3_ (Table 1) and with increasing duration of preservation period. For describing this relationship, a complex regression model (Equation (1)) was used giving more information about the effects of the experimental factors: feed matrix, moisture content, Na_2_SO_3_ addition and preservation duration. Here, the independent variables were constituted by the two last named factors. The results indicated that the higher moisture content of 30% as well as higher dosages of sodium sulfite applied were favorable for DON degradation. In the variants, MK 30% and MM 30% treated with 10 g Na_2_SO_3_ was already sufficient for a complete DON reduction after eight and three days of preservation time, respectively. In contrast, after the same time, variant MK and MM 14% achieved a DON reduction rate of 82% and 39%, respectively. Regarding the DON reduction after 10 min of mixing, the DON content was reduced by 88%–92% in high moisture variants in comparison to 72% and 37% by MK 14% and MM 14%, respectively. The further reduction of DON in the remaining preservation time averaged about 9.5%. The maximum reduction rate of low moisture and high Na_2_SO_3_ variants was 87% in MK 14% and only 40% in MM 14% after the whole preservation period. With low dosages of 1.25 g and 2.5 g Na_2_SO_3_, only marginal DON reduction was found (Table 1) and also the long preservation period could barely enhance the effect (Figure 1). Moreover, after 79 days for all variants except for MM 14% moisture, the concentration of DON slightly increased again. Regarding the dosage of 5 g Na_2_SO_3_ per kg, reduction rates of 25% and 91% were found for variants MM 14% and MM 30%, respectively, at the end of the preservation period. The corresponding recovery for variants MK 14% and MK 30% amounted to 79% and 80%, respectively. The estimation of the Na_2_SO_3_ dose necessary for reducing the DON concentration by 50% (Na_2_SO_3 1/2β_) was much lower in variant MK 30% than in MK 14%. The difference amounted to 4.37 g Na_2_SO_3_ per kg maize. In variants MM 14% and MM 30%, similar relationships were observed but differed more clearly (13.06 g Na_2_SO_3_ per kg maize). Furthermore, the time for the DON reduction of 50% (t_1/2α_) was calculated and confirmed with the previously stated results. For MM 30% and MK 30%, the terminal half-lives t_1/2α_ amounted 0.0103 days and 0.0077 days, which was less than 15 min, followed by MK 14% with 0.41 days (9 h 50 min). In contrast, for MM 14%, 50% of DON content was reduced after 9.05 days (Table 2).

### 2.3. DON Sulfonate Concentrations

**Figure 2:**
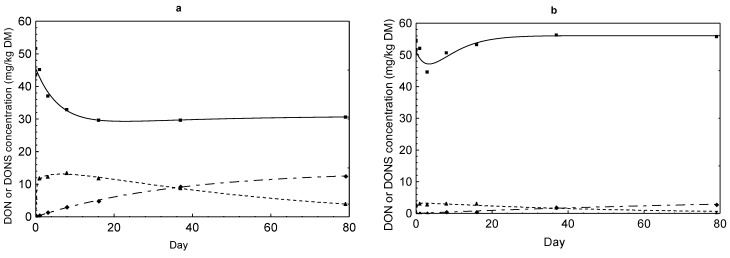

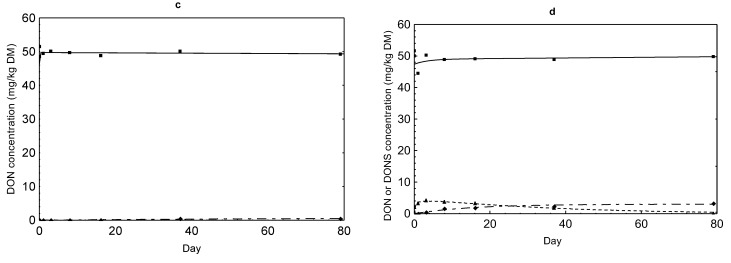
Comparison of the time courses of DON (full line marked with a square) and DONS 2 (dashed line of alternating short and long lines marked with a diamond) and DONS 3 (dashed line marked with a triangle) concentrations in maize preserved with 1.25 g Na_2_SO_3_ per kg in dependence on feed matrix (kernels = MK or meal = MM) and moisture content (14% or 30%): (**a**) MK 14%; (**b**) MK 30%; (**c**) MM 14%; (**d**) MM 30%.

**Figure 3:**
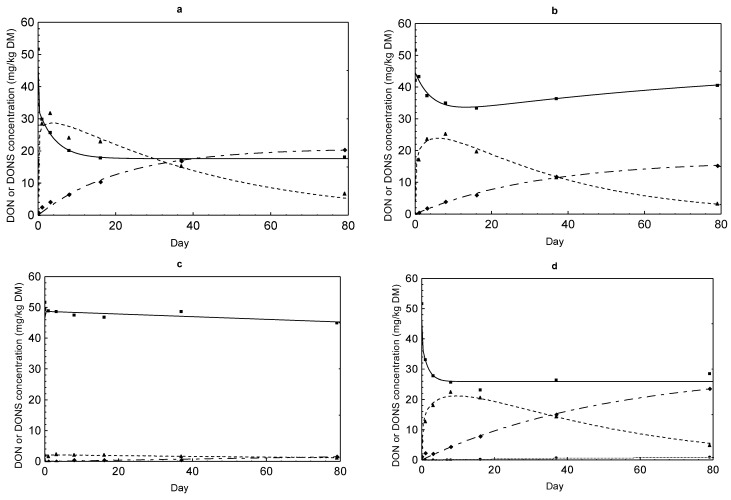
Comparison of the time courses of DON (full line marked with a square), DONS 1 (dotted line marked with a circle), 2 (dashed line of alternating short and long lines marked with a diamond) and 3 (dashed line marked with a triangle) concentrations in maize preserved with 2.5 g Na_2_SO_3_ per kg in dependence on feed matrix (MK or MM) and moisture content (14% or 30%): (**a**) MK 14%; (**b**) MK 30%; (**c**) MM 14%; (**d**) MM 30%.

**Figure 4:**
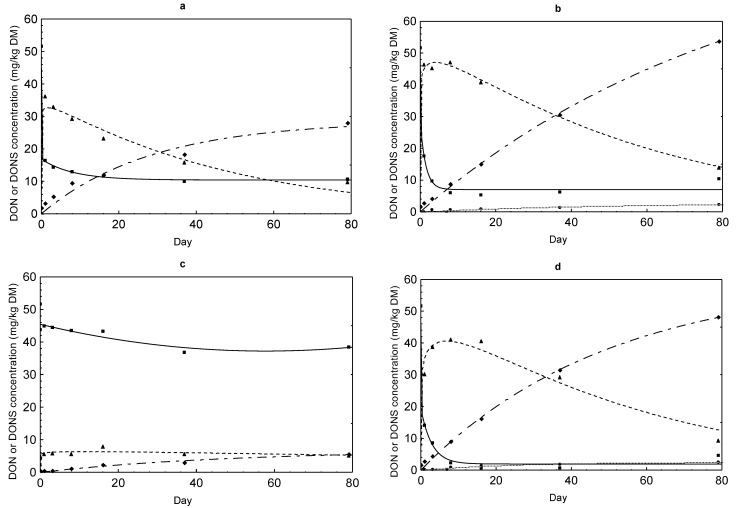
Comparison of the time courses of DON (full line marked with a square) and DONS 1 (dotted line marked with a circle), 2 (dashed line of alternating short and long lines marked with a diamond) and 3 (dashed line marked with a triangle) concentrations in maize preserved with 5 g Na_2_SO_3_ per kg in dependence on feed matrix (MK or MM) and moisture content (14% or 30%): (**a**) MK 14%; (**b**) MK 30%; (**c**) MM 14%; (**d**) MM 30%.

**Figure 5:**
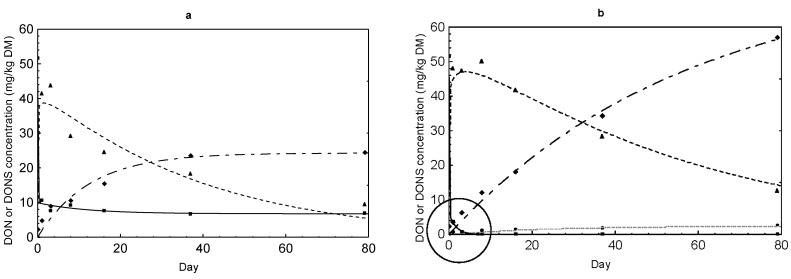

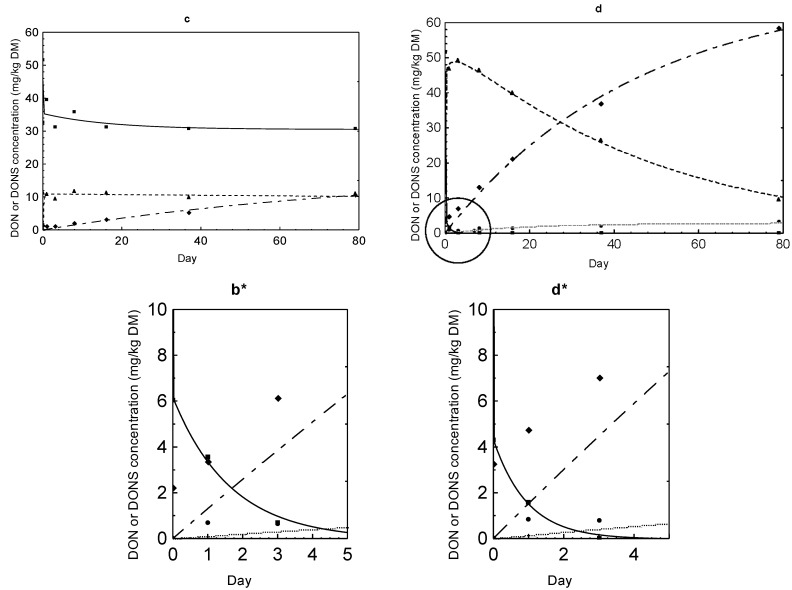
Comparison of the time courses of DON (full line marked with a square) and DONS 1 (dotted line marked with a circle), 2 (dashed line of alternating short and long lines marked with a diamond) and 3 (dashed line marked with a triangle) concentrations in maize preserved with 10 g Na_2_SO_3_ per kg in dependence on feed matrix (MK or MM) and moisture content (14% or 30%): (**a**) MK 14%; (**b**) MK 30%; (**c**) MM 14%; (**d**) MM 30%. Small graphics (b*,d*): the magnified area showed the initial decrease of DON concentration as well as the increase of DONS 1 and 2.

DON reduction was accompanied by a simultaneous formation of DONS 1, 2 and 3 (Figure 2, Figure 3, Figure 4 and Figure 5). The evolution of these derivatives apparently varied in dependence on Na_2_SO_3_ dose and other reaction conditions. DONS 1 was only rarely formed in the variants with 30% moisture, while DONS 2 and 3 were detected in almost every variant where Na_2_SO_3_ was supplemented. Of particular interest was the fact that DONS 3, which was quickly formed immediately after mixing, decreased continuously with increasing duration of preservation (Figure 4 and Figure 5). Concurrently with the decrease of DONS 3, the concentration of DONS 2 increased strongly (e.g., Figure 4b,d). At lower Na_2_SO_3_ additions, the time course of DONS 2 and 3 was similar but less pronounced (Figure 2 and Figure 3). The kinetics of DONS 1, 2 and 3 were described with two statistical models because of the difference in curve progression between DONS 3 and the two others. By means of the two equations, interested regression parameters could be derived (Table 3). On the one hand, the determination of y_max_ characterized the asymptotic value for DONS 1 and 2 at the end of the preservation duration and on the other hand the maximum concentration of DONS 3 occurred at time t_max_. The t_max_ corresponding upper limit of DONS 1 only was 2.83 mg/kg DM and 2.16 mg/kg DM for variants MM 30% and MK 30% whereas DONS 2 reached 57.75 mg/kg DM and 56.43 mg/kg DM for MM 30% and MK 30%, respectively. Compared with DONS 3, the maximum concentration of MM 30% and MK 30% already amounted to 48.87 mg/kg DM and 49.71 mg/kg DM after 2.24 days and 2.13 days, respectively. However, after reaching the highest concentration DONS 3 decreased up to preservation end. Hence, the content of DONS 3 after 79 days was 12.52 mg/kg DM and 9.40 mg/kg DM for MM 30% and MK 30%, respectively. Furthermore, the time t_1/2AUC_ (d) for DONS 3 was calculated, indicative of the time where the half of the total area under the curve (AUC) was achieved. Here, no differences between variants MK 14% and 30% as well as MM 30% existed, and t_1/2AUC_ was between 24.29 days and 39.75 days. In contrast, a range between 91 and 784 days was determined for MM 14%.

**Table 1:** Summary of important parameters of the preservation experiment: measured moisture content and pH value, microbial status and remaining deoxynivalenol (DON) and DON sulfonates (DONS) concentration of maize initially contaminated with 51.6 mg DON/kg dry matter (DM).

Variant	Feed Matrix	Na_2_SO_3_^a^ (g/kg)	Measured Moisture (%) (*n* = 7) ^b^	pH (Day 79)	Yeasts (Day 37, log CFU/g)	Yeasts (Day 79, log CFU/g)	Moulds (Day 37, log CFU/g)	Moulds (Day 79, log CFU/g)	DON Reduction (Day 79; %)	DONS 1 (Day 79, % of Initial DON)	DONS 2 (Day 79, % of Initial DON)	DONS 3 (Day 79, % of Initial DON)	Recovery (DON+DONS 1, 2 and 3, Day 79, % of Initial DON)
1	MK ^1^	0	14.6 ± 0.3	4.57	n.d.	n.d.	2.12	n.d.	7	0	0	0	93
2	MK	1.25	14.3 ± 0.5	4.76	n.d.	n.d.	n.d.	n.d.	41	0	24	8	91
3	MK	2.5	14.7 ± 0.6	4.80	n.d.	1.51	n.d.	n.d.	65	0	40	13	88
4	MK	5	14.7 ± 0.6	4.74	n.d.	n.d.	n.d.	1.81	79	0	54	19	94
5	MK	10	14.6 ± 0.7	4.87	n.d.	n.d.	n.d.	n.d.	87	0	48	18	79
6	MK	0	30.4 ± 0.6	4.63	n.d.	n.d.	n.d.	1.51	0	0	0	0	100
7	MK	1.25	30.5 ± 1.0	4.64	n.d.	n.d.	n.d.	n.d.	0	0	6	0	106
8	MK	2.5	31.1 ± 1.0	4.66	n.d.	n.d.	n.d.	n.d.	21	0	30	6	115
9	MK	5	31.2 ± 1.1	4.70	n.d.	n.d.	n.d.	n.d.	80	5	104	27	156
10	MK	10	30.0 ± 0.8	4.95	n.d.	n.d.	n.d.	n.d.	100	5	110	24	139
11	MM ^2^	0	13.0 ± 0.2	4.55	n.d.	1.81	2.63	1.81	0	0	0	0	100
12	MM	1.25	13.0 ± 0.2	4.57	n.d.	n.d.	n.d.	1.51	4	0	1	0	97
13	MM	2.5	12.9 ± 0.2	4.61	n.d.	n.d.	2.0	n.d.	13	0	3	2	92
14	MM	5	13.3 ± 2.3	4.72	n.d.	n.d.	n.d.	n.d.	25	0	11	10	96
15	MM	10	13.0 ± 0.3	4.85	n.d.	1.51	n.d.	n.d.	40	0	21	21	102
16	MM	0	29.5 ± 0.4	4.56	n.d.	n.d.	n.d.	n.d.	0	0	0	0	100
17	MM	1.25	29.3 ± 0.5	4.59	n.d.	1.81	n.d.	n.d.	3	0	6	0	103
18	MM	2.5	29.3 ± 0.4	4.60	n.d.	n.d.	n.d.	1.51	45	2	46	9	112
19	MM	5	29.2 ± 0.3	4.71	n.d.	n.d.	n.d.	n.d.	91	5	93	18	125
20	MM	10	29.2 ± 0.3	4.86	n.d.	n.d.	n.d.	n.d.	100	6	113	18	137

n.d.: Not detectable; ^1^ MK = maize kernels, ^2^ MM = maize meal; ^a^ Sodium sulfite (Na_2_SO_3_), BASF SE, 67056 Ludwigshafen, Charge. 50700456P0; ^b^ mean values with standard deviation.

**Table 2:** Summary of multiple exponential regressions of preservation duration (t) and of sodium sulfite on deoxynivalenol (DON) concentration of maize according to Equation (1) ^a^.

Moisture Content (%)	14	30	14	30
Feed Matrix	MK ^1^	MK	MM ^2^	MM
A	22.88	100	3.43	−57.96
α	1.7	89.77	0.08	67.62
B	23.8	−49.12	48.13	109
β1	0	496	13.07	30.14
β2	−808	−1.03	−1.29	305
β3	−2.72	0.32	−0.16	0.001
C	20.37	61.77	50.54	55.57
γ1	0.1	0.26	0.04	0.3
γ2	2.21	9.57	10.73	7.74
t_1/2α_ (d)	0.41	0.0077	9.05	0.0103
Na_2_SO_3 1/2γ1_ (g Na_2_SO_3_/kg)	7.06	2.69	15.41	2.35
RSD (mg/kg)	4.29	6.88	1.91	6.89
r^2^	0.971	0.96	0.969	0.958

RSD: Residual Standard Deviation; ^1^ MK = maize kernels, ^2^ MM = maize meal; ^a^ DON (mg/kg DM) = A × e^−α × t^ + B × e^−β1 × t × (1 + β2 × (1 − e^−β3 × Na_2_SO_3_^))^ + C × e^−γ1 × Na_2_SO_3_ × (1 + e^−γ2 × t^)^ where the first term presents the effect of time following the initial preservation phase on DON concentration and allows calculating a terminal half-life (t_1/2α_). The second and third term consider the interactions between time and Na_2_SO_3_ addition. The sum of A + B equals the regressively estimated initial DON concentration for time = 0. Parameter C is the DON concentration for infinite time and a zero Na_2_SO_3_ concentration. α, β1, β2, β3, γ_1_ and γ_2_ are the corresponding rate constants for the first, second and third terms, respectively.

**Table 3:** Summary of regression parameters of different DON sulfonates (DONS) concentration of maize during the preservation period according to Equation (2) for DONS 1 and DONS 2 ^a^ as well as Equation (3) for DONS 3 ^b^.

Variants		Na_2_SO_3_ Addition	DONS Compound			Model	Regression Parameters ± SE			y_max_	t_max_ (d)	t _1/2 AUC_ (d)	r^2^	RSD
ID	Matrix + Moisture		DONS 1	DONS 2	DONS 3		a	b	c					
V9	MK ^1^ 30%	5	x	-	-	1	2.46 ± 1.06	0.03 ± 0.02	-	2.26	-	-	0.941	0.30
V10	MK 30%	10	x	-	-	1	2.16 ± 0.82	0.08 ± 0.04	-	2.16	-	-	0.946	0.30
V18	MM ^2^ 30%	2.5	x	-	-	1	1.26 ± 0.54	0.02 ± 0.01	-	0.96	-	-	0.978	0.09
V19	MM 30%	5	x	-	-	1	2.47 ± 0.25	0.05 ± 0.01	-	2.42	-	-	0.980	0.19
V20	MM 30%	10	x	-	-	1	2.86 ± 0.68	0.06 ± 0.03	-	2.83	-	-	0.876	0.51
V2	MK 14%	1.25	-	x	-	1	14.01 ± 0.53	0.03 ± 0.002	-	12.53	-	-	0.999	0.27
V3	MK 14%	2.5	-	x	-	1	20.81 ± 1.20	0.05 ± 0.01	-	20.28	-	-	0.992	1.02
V4	MK 14%	5	-	x	-	1	28.61 ± 3.55	0.04 ± 0.01	-	26.85	-	-	0.978	2.14
V5	MK 14%	10	-	x	-	1	24.28 ± 2.17	0.08 ± 0.02	-	24.24	-	-	0.971	2.37
V7	MK 30%	1.25	-	x	-	1	5.50 ± 1.83	0.01 ± 0.004	-	2.91	-	-	0.993	0.14
V8	MK 30%	2.5	-	x	-	1	16.83 ± 0.51	0.03 ± 0.002	-	15.36	-	-	0.999	0.29
V9	MK 30%	5	-	x	-	1	89.06 ± 10.57	0.01 ± 0.002	-	53.66	-	-	0.998	1.15
V10	MK 30%	10	-	x	-	1	75.41 ± 8.70	0.02 ± 0.003	-	56.43	-	-	0.996	1.94
V12	MM 14%	1.25	-	x	-	1	0.74 ± 0.55	0.01 ± 0.02	-	0.49	-	-	0.907	0.09
V13	MM 14%	2.5	-	x	-	1	39.55 ± 89.70	0.0005 ± 0.001	-	1.50	-	-	0.953	0.18
V14	MM 14%	5	-	x	-	1	6.85 ± 1.35	0.02 ± 0.01	-	5.43	-	-	0.984	0.36
V15	MM 14%	10	-	x	-	1	18.10 ± 8.83	0.01 ± 0.01	-	10.51	-	-	0.975	0.83
V17	MM 30%	1.25	-	x	-	1	3.00 ± 0.33	0.07 ± 0.02	-	2.98	-	-	0.977	0.29
V18	MM 30%	2.5	-	x	-	1	29.93 ± 3.25	0.02 ± 0.004	-	23.46	-	-	0.995	0.85
V19	MM 30%	5	-	x	-	1	60.44 ± 3.40	0.02 ± 0.002	-	48.04	-	-	0.999	0.99
V20	MM 30%	10	-	x	-	1	70.11 ± 7.46	0.02 ± 0.004	-	57.75	-	-	0.994	2.44
V2	MK 14%	1.25	-	-	x	2	11.61 ± 0.22	0.14 ± 0.01	0.02 ± 0.001	13.13	6.52	38.59	0.998	0.38
V3	MK 14%	2.5	-	-	x	2	27.91 ± 1.43	0.09 ± 0.02	0.03 ± 0.005	28.68	3.63	29.99	0.983	2.37
V4	MK 14%	5	-	-	x	2	33.28 ± 1.58	0.04 ± 0.02	0.02 ± 0.004	32.66	1.64	32.55	0.984	2.58
V5	MK 14%	10	-	-	x	2	39.65 ± 3.10	0.04 ± 0.03	0.03 ± 0.01	38.70	1.49	26.98	0.965	4.68
V7	MK 30%	1.25	-	-	x	2	3.25 ± 0.24	0.04 ± 0.03	0.02 ± 0.01	3.20	1.93	31.94	0.962	0.43
V8	MK 30%	2.5	-	-	x	2	20.20 ± 0.96	0.21 ± 0.03	0.04 ± 0.004	23.88	5.99	25.46	0.991	1.47
V9	MK 30%	5	-	-	x	2	45.77 ± 0.71	0.07 ± 0.01	0.02 ± 0.001	47.07	3.96	40.51	0.998	1.27
V10	MK 30%	10	-	-	x	2	50.21 ± 1.10	0.04 ± 0.01	0.02 ± 0.002	49.71	2.13	38.22	0.996	1.94
V13	MM 14%	2.5	-	-	x	2	2.10 ± 0.15	0.01 ± 0.03	0.01 ± 0.004	2.09	1.50	90.53	0.949	0.28
V14	MM 14%	5	-	-	x	2	5.95 ± 0.38	0.05 ± 0.03	0.004 ± 0.003	6.36	11.21	177.48	0.956	0.78
V15	MM 14%	10	-	-	x	2	10.91 ± 0.47	0.00001 ± 0.02	0.0009 ± 0.002	10.91	0.01	783.60	0.977	0.98
V17	MM 30%	1.25	-	-	x	2	3.75 ± 0.22	0.13 ± 0.03	0.03 ± 0.01	3.93	3.88	24.29	0.982	0.35
V18	MM 30%	2.5	-	-	x	2	15.32 ± 1.23	0.26 ± 0.05	0.03 ± 0.005	21.20	9.52	34.77	0.984	0.98
V19	MM 30%	5	-	-	x	2	35.65 ± 1.92	0.14 ± 0.03	0.02 ± 0.003	40.59	6.79	39.75	0.982	3.34
V20	MM 30%	10	-	-	x	2	49.34 ± 0.54	0.05 ± 0.004	0.02 ± 0.001	48.87	2.24	33.06	0.999	0.93

RSD: Residual Standard Deviation, ^1^ MK = maize kernels, ^2^ MM = maize meal; ^a^ y = α × (1 − e^(−b × t)^) where y = concentration of DONS 1 or DONS 2 (mg/kg DM), t = time, a and b = regression coefficients. When time converges to infinity the concentration of DONS 1 or DONS 2 tends towards the asymptotic value “a”. The coefficient b indicates the increase of the concentration; ^b^ y = α × t^b^ × e^(−c × t)^ where y = concentration of DONS 3 (mg/kg DM), t = time and a to c = regression coefficients. Based on the estimated coefficients further parameters could be determined: t_max_ = b/c (time at maximum concentration, d = days), y_max_ = α × t_max_^b^ × e^c × tmax^ (maximum concentration, mg/kg DM), t_1/2AUC_ = gammainv (0.5, b+1, 1/c) (time which corresponds to the half of the AUC, h).

## 3. Discussion

In the present experiment, SS was tested as an alternative to SBS for the reduction of DON during the wet preservation of maize. In general, by using sodium sulfite for wet preservation mediated DON reduction, the kinetics followed a similar pattern compared to using SBS as described in a previous study by Dänicke *et al.* (2009) [10]. According to tests of Schwartz *et al.* (2013) [11], the formation of three different DON sulfonates accompanied the degradation of DON. In general, the moisture content might play an important role for the reaction progress because of a consistently more efficient DON reduction. The advantage of higher moisture content levels was also confirmed by Dänicke *et al.* (2009) [10], although the moisture content levels of 13% and 15% in triticale kernels used in their study were lower than in the present investigation. The estimation of time for 50% reduction of DON (Equation (1)) differed strongly between both moisture content levels. In order to verify the influence of the parameter preservation time on the reaction of DON and sodium sulfite, a long period of about 79 days was chosen and carefully studied. It became clear that the degradation of DON took place very quickly if the dose amounted to ≥5 g/kg and in combination with higher moisture content. In maize meal, large differences between the reaction time were demonstrated and amounted to 9.05 days and 0.0103 days (15 min) for 14% and 30% moisture, respectively. Maize kernels required only 0.41 days (9 h 50 min) and 0.0077 days (11 min) at the same moisture content levels. Much longer times of 2.8 days and 2.0 days were required for triticale preservation at 13% and 15% moisture, respectively, as reported by Dänicke *et al.* (2009) [10]. Therefore, the interaction between moisture and feed matrix was an interesting means to find out the optimum conditions for DON reduction (Figure 1). The 3D models of kernels and meal strongly differed at 14% moisture whereby the kernel structure might be advantageous. Here, the DON reduction amounted to 40% compared to 87% for MM 14% and MK 14%, respectively. At 30% moisture, the difference disappeared and variant MM 30% outnumbered even further the reduction rates and reaction velocity compared with kernel variants (Table 2). In this case, both MM 30% and MK 30% achieved 100% DON reduction. The feed structure, including factors like surface condition and particle sizes, seemed to be an important fact for the reaction of DON contaminated cereal with the supplement. One hypothesis explaining the inefficiency of the variant MM 14% might lie in the surface enlargement achieved by the crushing. As a result of the grinding process, the contact area expanded very much so that many small grain particles were formed. Thus, less freely available water was present per unit of surface for the reaction. In addition, given the low available water content, an uneven distribution of sodium sulfite could be the result. Therefore, ground cereals with low moisture content required a higher addition of sodium sulfite in comparison to the meal or kernel variants with higher moisture content. Another explanation could be the distribution of DON in maize kernels. According to Koehler (1942) [14], *Fusarium* infection occurs first on the tip cap and passes into the pericarp, so that the kernel stalks and the superficial regions are particularly highly contaminated (83%–99% infection rate). Depending on how far the fungus penetrated into the grain interior, mycotoxin contamination of the endosperm would be possible (49%–70% infection rate). Also, Young *et al.* (1984) [15] noted that a high DON concentration was found in the aleurone and outer husk layer which can be found in the bran fraction and micro particles through the milling process, and on the other hand in the flour fraction less DON was detectable. These aspects could explain why the DON reduction of MM 14% was lower. Due to the low proportion of free water, sodium sulfite might be inadequately distributed in ground particles, so that no satisfying DON reduction occurred. This is in contrast to MK 14% where the Na_2_SO_3_-water mixture was sufficient to achieve 87% DON degradation. However, variants with higher moisture content were more efficient in inducing DON reduction rates by up to 100%. These results were in accordance to those by Dänicke *et al.* (2009) [10], where similar kinetics was observed. Here, the DON reduction course followed a bi-exponential fashion and became obvious when SBS was ≥3g/kg, compared to ≥2.5 g Na_2_SO_3_/kg in this study. To obtain a maximum decontamination of DON in maize, the dosage of sodium sulfite supplemented was the “tip on the scale”. The highest dose of 10 g/kg was most effective in conjunction with 30% moisture. In comparison to adding 5 g Na_2_SO_3_, the DON reduction rate amounted to 80% and 91% for MK 30% and MM 30% after 79 days. Lower dosages of sodium sulfite had no consistent results. The hypothesis that higher moisture content could be used to reduce the chemical amount used for a significant DON reduction could be confirmed. This was best illustrated by the dose necessary to reduce the DON concentration by 50%. Only 2.35 g and 2.69 g Na_2_SO_3_ were needed for MM and MK 30%, respectively, corresponding to only a few minutes’ reaction time. In contrast, additions of 7.06 g and 15.41 g were necessary to halve the initial DON content in low moisture variants (Table 2). All estimations could be substantiated by the high goodness of fit (r^2^ = 0.958–0.971). Consequently, in order to minimize the amount of sodium sulfite, high moisture content was required for both feed matrices. Compared to Dänicke *et al.* (2009) [10], SBS additions between 3.34 g und 1.83 g SBS were used to reduce the DON concentration by 50% in triticale kernels. Taken together, the comparison of maize kernels and triticale kernels as well as sodium sulfite and SBS demonstrated some differences. It seemed that the SBS quantity used was lower at higher moisture content but the mode of reaction of Na_2_SO_3_ proceeded faster in maize kernels.

### DON Sulfonate Concentrations

Due to the reduction of DON, three different DON sulfonates were formed resulting from the reaction of DON with sodium sulfite. It was previously shown that the reaction of DON with SBS takes place at C10 [6,16]. Recently, Schwartz *et al.* (2013) [11] discovered formation of three DON sulfonates of which DONS 1 is characterized by loss of the epoxide group and DONS 2 by a formation of a hemiketal between C8 and C15. DONS 3 exists, in contrast, as a mixture of the two compounds hemiketal and keto which form in equilibrium and differs from DONS 2 in the stereochemistry at C9. Next to the decrease of the DON content, a concomitant increase of DON sulfonates took place (Figure 2, Figure 3, Figure 4 and Figure 5). The reaction efficiency, dependent on sodium sulfite addition and moisture content, was crucial for the extent of DONS formation. As first described for barley flour and maize treated with 5 g/kg sodium sulfite at moisture contents of 14% and 30% for seven days, the formation pattern of DONS 1, 2 and 3 turned out to be very different [12]. By adding 1.25 g and 2.5 g Na_2_SO_3_, only marginal DONS 2 and 3 concentrations could be found in maize (Figure 2 and Figure 3). Additions of ≥5 g/kg generated a steep initial increase of DONS 3 and a steady increase of DONS 2 and, to a smaller extent, DONS 1 (Figure 4 and Figure 5). For example, in variant 20 (Table 1), also with regard to y_max_ (Table 3), the maximum concentration of 48.87 mg DONS 3/kg DM after 2.24 days decreased to approximately 9.40 mg DONS 3/kg DM after 79 days. Compared to DONS 2, the concentration increased from some 5.88–57.75 mg/kg DM after 79 days. Here, DONS 1 played a minor role. In retrospect, the results showed that the preservation time was more decisive for the observation and the progress of the DONS formation. The DONS 2 to DONS 3 ratio changed for the benefit of DONS 2 at the end of the preservation period. The type of DONS formed in the treated feedstuff was influenced by the key factor pH value [11]. In the preserved maize variants, the pH value varied between 4.55 and 4.95 which favored the DONS 3 formation, because this metabolite is formed exclusively under acidic to neutral conditions. Furthermore, Schwartz-Zimmermann *et al.* (2014) [17] described DONS 3 converts to DONS 2 and back to DON at pH values >6 with concentration values changing over the storage time. An explanation is the instability of DONS 3 due to its structure [11]. Thus, the slight increase in DON concentration at the end of the preservation time could also be traced back to the DONS 3 conversion to DON (e.g., Figure 2a, Figure 3b,d and Figure 4b,d). When alkaline pH ranges would prevail, DONS 1 and 2 would be the dominant reaction products and back formation to DON would only be observed under strong alkaline conditions (pH>10) or at a high temperature [11]. From a practical point of view, these discoveries could play a decisive role because of the wide pH ranges of 2.2 up to 7.8 prevailing along the digestive tract [18]. It could be assumed that dietary DONS 3 is quickly restructured to DONS 2 or converted to DON in the digestive tract. All other substances are supposed to be stable. According to that, high DONS 2 and low DONS 3 concentrations were favorable for a later use. Therefore, a longer preservation time should be kept to ensure a specific DONS pattern in acidified feedstuff. However, before testing these assumptions in appropriate feeding trials, the toxicity of the different DON sulfonates had to be clarified. The loss of toxicity ultimately was the main objective of the decontamination process. In previous toxicity studies of Beyer *et al.* (2010) [16] and Dänicke *et al.* (2010) [19], it was shown that DONS was non- or less toxic than DON tested on porcine cells (IPEC) and humane cell lines. However, the new insights of Schwartz *et al.* (2013) [11] revealed that not only one DONS, but three DONS compounds were proven. To determine the toxicity of them, several approaches were used. The toxicity of DONS 1, 2 and 3 on the ribosome and the effects on the viability of intestinal porcine epithelial cells (IPEC-J2) were tested as well as a yeast bioassay was performed [12]. All in all, the results of the different assays showed that DONS 1 was non-toxic and a lesser inhibitory effect was proven for DONS 2 and 3 in comparison to DON. In our interest, the IPEC-J2 cells viability was important to know because pigs are the most sensitive species. Here, DONS 1 did not affect the viability, and DONS 2 was less toxic by the factor of 77 than DON. DONS 3 was unstable in the assay and could not be tested. In the ribosome assay, DONS 3 followed a similar trend as DONS 2.

Considering the decontamination demands, the use of sodium sulfite in preserved feed might be an effective DON decontaminating procedure which could be used at the farm level. In order to establish this detoxification method in practice, possibilities for feed treatment and feed storage must be explored, and a cost-benefit analysis should be completed [2].

## 4. Experimental Section

### 4.1. Preservation Experiment

#### 4.1.1. Contaminated Maize

For the preservation experiment, contaminated maize was used which had previously been cultivated on the experimental field of the Institute of Animal Nutrition of the Friedrich-Loeffler Institute in Braunschweig, Germany, and had been inoculated with spores of *Fusarium graminearum* at a concentration of 200,000 spores/mL in the lactic ripeness stage. An amount of 0.5 mL of the infection solution had been applied directly into the cob through the opening of the husk [20]. Two and a half months later, the contaminated maize cobs were harvested.

#### 4.1.2. Experimental Design

The preservation experiment included the four following factors:

Two different feed structures: maize kernels (MK) and maize grain meal (MM)
Moisture contents of 14% and 30%
Na_2_SO_3_ additions of 0, 1.25, 2.5, 5 and 10 g/kg maize
Preservation duration of 0, 0.007 (10 min), 1, 3, 8, 16, 37 and 79 days.

The factors were combined in a multi-factorial design. Therefore, the experiment started with 2 (feed structure) × 2 (moisture contents) × 5 (sodium sulfite additions) variants, resulting in a total of 20 variants which were sampled seven times each (Table 4). The contaminated maize with an initial DON concentration of 51.6 mg/kg dry matter (DM) served as the basic material for all variants (day 0) and the first sample was taken immediately after a 10-min mixing of the components (0.007 days). This was followed by further sampling at defined time points.

**Table 4:** Experimental design with all combinations of feed matrices and moisture contents with increasing Na_2_SO_3_ additions and a constant addition of 15 g propionic acid/kg.

Variants	Feed Matrix	Planned Moisture Content (%)	Sodium Sulfite (g/kg Maize)
1	MK = Maize kernels	14	0
2	MK	14	1.25
3	MK	14	2.5
4	MK	14	5
5	MK	14	10
6	MK	30	0
7	MK	30	1.25
8	MK	30	2.5
9	MK	30	5
10	MK	30	10
11	MM = Maize meal	14	0
12	MM	14	1.25
13	MM	14	2.5
14	MM	14	5
15	MM	14	10
16	MM	30	0
17	MM	30	1.25
18	MM	30	2.5
19	MM	30	5
20	MM	30	10

#### 4.1.3. Procedures and Sample Preparation

For the preservation experiment, the harvested and dried contaminated maize grain was divided into two subsets. A part of whole maize kernels (MK) was used unground while the other was ground to pass through a 3 mm screen (maize meal, MM) before the following treatments. Subsequently, the individual variants were mixed with the chemicals and water for 10 min using a closable mixer. The required amount of water was calculated based on the residual moisture content of maize in order to adjust variants to be preserved to targeted moisture contents of 14% or 30%. All variants were generally preserved with 15 g/kg of propionic acid in order to prevent microbial spoilage. After mixing, the maize was filled into preserving jars, duplicate samples of approximately 500 g for each sampling time. A total of 280 jars were filled.

The first sample was taken after 10 min mixing and stored in the freezer at −18 °C until analysis. Of all variants, the jars were tightly sealed and stored in a climatic chamber at 18 °C for the certain preservation periods (1, 3, 8, 16, 37 and 79 days). At the specified sampling time points, two jars of each variant were taken, mixed, filled into a sample bag and frozen at −18 °C. Moreover, sub-samples were collected for determination of the dry matter content. On 37th and 79th day, samples were also checked for their microbiological status. After completion of the experiment, a total of 140 samples were available for further laboratory analysis. Before analysis, samples were milled to pass through a 1 mm screen.

### 4.2. Analyses

#### 4.2.1. Mycotoxins

DON and DON sulfonates in maize were determined by a RP-UHPLC-MS/MS (reversed phase ultra-high performance liquid chromatography coupled with tandem mass spectrometry) based method according to Schwartz-Zimmermann *et al.* (2014) [17]. The method consisted of two extractions of 1 g of maize with 10 mL each of acidified extraction solvent, 1 + 9 dilution and subsequent RP-UHPLC-MS/MS analysis on an Agilent 1290 series UHPLC system coupled to a 4000 QTrap mass spectrometer (AB Sciex, Foster City, CA, USA) in a negative ion mode after electrospray ionization (ESI). The mass spectrometric detection used the following transitions: DONS 1 and DONS 3: DP −91 V, quant: 377.1→79.9 (CE −88 eV), qual: 377.1→81.0 (CE −60 eV); DONS 2: DP −70 V, quant: 377.1→81.0 (CE −60 eV), qual: 377.1→79.9 (CE −88 eV); DON: DP −70 V, quant: 341.1→45.0 (CE −30 eV), qual: 341.1→265.0 (CE −14 eV). Apparent recoveries were between 94% and 108% for all analytes. LOQs were 0.46 mg/kg for DONS 1, 0.37 mg/kg for DONS 2, 0.56 mg/kg for DONS 3 and 1.8 mg/kg for DON in maize and the relative standard deviation of quadruplicate work-up and analysis was <5%.

#### 4.2.2. pH Value and Moisture Content

The pH value of the individual maize samples was determined at the time of laboratory analysis using a pH meter (WTW GmbH, Weilheim, Germany). For this purpose, each sample was mixed with distilled water at the ratio of 1:4, shaken and immediately measured.

Determination of the dry matter content was carried out during the preservation experiment on the specified sampling time points, and again at the time of laboratory analysis. Samples were dried for 3 h at 105 °C and were weighed before and after. From the weight difference, the dry matter was calculated by the method of the Verband Deutscher Landwirtschaftlicher Forschungs- und Untersuchungsanstalten (VDLUFA) [21].

#### 4.2.3. Microbiological Status

The microbial status of the preserved maize samples was determined at 37th and 79th day of preservation duration by counting yeasts and moulds (CFU).

To determine the colony count, a surface spread method was applied on modified malt extract agar (Malt extract: No. 5391, MERCK, supplemented with 3 g peptone from soy meal; No. 7212, MERCK), autoclaved at 121 °C for 15 min and finally adjusted to pH 3.5 by using 10% lactic acid at 50 °C just prior to pouring the Petri dishes [10]. Maize samples were ground to meal using a cross hammer mill for 60 s with short interruptions to avoid heating above 35 °C. Thirty grams of the meal were weighted into a Stomacher-bag, suspended in 270 mL of sterile demineralized water and pulped for 2 min in a Stomacher-lab blender. From the suspension, the 10^−1^ dilution was prepared, and basing on previous experiments, 100 µL of this dilution was inoculated on the pre-dried agar surface in triplicate by using a Drigalski spatula (VWR, Darmstadt, Germany). Yeast and mould colonies were enumerated after three days of aerobic incubation at 30 °C. The limit of detection of this method amounted to 10 CFU/g maize.

The results of the microbiological examination (Table 1) indicated that in 11 of 40 samples contained either yeast or mould colonies while one sample was contaminated by both at the same time. After 37 days, only mould colonies were detected in three variants whereby the kernel and meal variant without sodium sulfite addition at 14% moisture were affected. At the end of the preservation, both yeasts and moulds were detected but the counts were below the limit of detection (2 log CFU/g). The yeasts or moulds colonies seemed to occur more often at the lower moisture content, so that a dependence on these factors could not be excluded. Nevertheless, the preservation success was confirmed. 

### 4.3. Statistics

The measured DON concentrations were evaluated regressively as described by [10]. Here, the data sets belonging to each of the planned moisture content levels (14% and 30%) and each of the two feed matrices were grouped together in a complex regression model which described the relationship between Na_2_SO_3_ dosage (0, 1.25, 2.5, 5 and 10 g/kg maize) and time (preservation duration) as per Equation (1):

DON (mg/kg DM) = A × e^−α × t^ + B × e^−β1 × t × (1 + β2 × (1 − e^−β3 × Na_2_SO_3_^))^ + C × e^−γ1 × Na_2_SO_3_ × (1 − e^−γ2 × t^)^ (1)

In this regressive evaluation, the first term describes the effect of time following the initial preservation phase on DON concentration and allows calculating a terminal half-life (t_1/2α_). The second and third term consider the interactions between time and Na_2_SO_3_ dosage. The steep initial DON reduction due to Na_2_SO_3_ addition is described by the second term and implies that the corresponding half-life is not a constant but depends on Na_2_SO_3_ dosage in an exponential manner. When time converges to infinity, the DON concentration depends solely on the Na_2_SO_3_ concentration which is characterized in the third term. It further enables the derivation of the Na_2_SO_3_ concentration necessary to reduce the DON concentration by 50% (Na_2_SO_3__1/2β_). Furthermore, the sum of A + B equals the regressively estimated initial DON concentration for time = 0 while C is the DON concentration for infinite time and a zero Na_2_SO_3_ concentration. The corresponding rate constants α, β1, β2, β3, γ_1_ and γ_2_ are allocated to the first, second and third term, respectively. Moisture content, pH value and yeast and mould colonies were evaluated descriptively. All statistics were carried out using the Statistica for the Windows™ operating system (StatSoft Inc., Tulsa, OK, USA, 2011).

For the statistical analysis of DONS 1, 2 and 3, two different models were used because of the differences in the kinetic behavior of the corresponding concentrations over time. The DONS 3 kinetics obviously differed from DONS 1 and 2. Therefore, these two DON metabolites were described as follows:

y = α × (1 − e^(−b × t)^) (2)

where y = concentration of DONS 1 or DONS 2 (mg/kg DM), t = time, a and b = regression coefficients. When time converges to infinity the concentration of DONS 1 or DONS 2 tends towards the asymptotic value “a”. The coefficient b indicates the increase of the concentration.

The kinetic of DONS 3 included first a steep initial increase to maximum concentration followed by a gradual decline. This course could be described by the Equation (3):

y = α × t^b^ × e^(−c × t)^ (3)

where y = concentration of DONS 3 (mg/kg DM), t = time, a to c = regression coefficients. Based on the estimated coefficients, further parameters could be determined:

t_max_ = b/c (time at maximum concentration, d = days)
y_max_ = α × t_max_^b^ × e^c × tmax^ (maximum concentration, mg/kg DM)
t_1/2AUC_ = gammainv (0.5, b+1, 1/c) (time which corresponds to the half of the AUC, h).

## 5. Conclusions

The wet preservation method with sodium sulfite demonstrated its detoxification potential for highly DON contaminated maize. Higher moisture content levels and Na_2_SO_3_ dosages greater than 2.5 g/kg had an advantageous effect on the DON reduction. The feed matrix markedly influenced the detoxification efficacy, especially at low moisture contents. Treatment with sodium sulfite potentially offers an effective way to detoxify DON contaminated maize products to be used as feedstuff. However, at lower moisture content, higher amounts of sodium sulfite are required for an efficient DON reduction. Based on the results of this study, further investigations are required with regard to the effectivity in feeding trails with pigs wherein open questions about their acceptance, absorption, distribution, metabolism and excretion in pigs shall be answered. Moreover, the *in vivo* stability of the various forms of DONS has to be examined. Additionally to the previous toxicity tests, a MTT (methylthiazolium)-assay with target cells like porcine peripheral blood mononuclear cells (PBMC) could be necessary wherein, besides the DON and DON sulfonates, also the influence of sodium sulfite is clarified.
